# Supplementary material for: Investigating the Differential Circulating microRNA Expression in Adolescent Females with Severe Idiopathic Scoliosis: A Proof-of-Concept Observational Clinical Study
Source: Int J Mol Sci. 2024 Jan 1;25(1):570. doi: 10.3390/ijms25010570 (PMC10779108; doi:10.3390/ijms25010570)
Supplement: Supplementary file 1 [file ijms-25-00570-s001.zip › Supplementary Caption Raimondi L et al.pdf]

**Supplementary Figure 1.** A. Heat map diagram shows cluster analysis of differentially expressed miRNAs. The colors represent the expression values of differentially expressed miRNAs. Sample names are listed in the horizontal axis. B. PCA Analysis of differentially expressed miRNAs in AIS patients and HC.

**Supplementary Figure 2.** A. Heat map diagram shows cluster analysis of differentially expressed miRNAs. The colors represent the expression values of differentially expressed miRNAs. Sample names are listed in the horizontal axis. B. PCA Analysis of differentially expressed miRNAs in severe female AIS patients and female HC.

**Supplementary Figure 3.** Heat map diagram shows cluster analysis of differentially expressed miRNAs. The colors represent the expression values of differentially expressed miRNAs. Sample names are listed in the horizontal axis. Axis represents the clustering of miRNAs. A. Heat map analysis of circulating miRNAs in moderate AIS vs HC. B. Heat map analysis of circulating miRNAs in severe male AIS vs males HC. C. PCA plot analysis of differentially expressed miRNAs in moderate AIS vs HC. D. PCA plot analysis of differentially expressed miRNAs in severe male AIS vs males HC.

**Supplementary Table 1.** Demographic and clinical data of 20 AIS patients.

**Supplementary Table 2.** Circulating miRNAs up- and down-regulated in plasma of AIS patients versus plasma of HC.

**Supplementary Table 3.** Circulating miRNAs up- and down-regulated in plasma of severe female AIS patients versus plasma of female HC.

**Supplementary Table 4.** Proteomic Data Analysis
