# Supplementary material for: Investigating the Differential Circulating microRNA Expression in Adolescent Females with Severe Idiopathic Scoliosis: A Proof-of-Concept Observational Clinical Study
Source: Int J Mol Sci. 2024 Jan 1;25(1):570. doi: 10.3390/ijms25010570 (PMC10779108; doi:10.3390/ijms25010570)
Supplement: Supplementary file 1 [file ijms-25-00570-s001.zip › Supplementary Figure 2 REVISED.pdf]

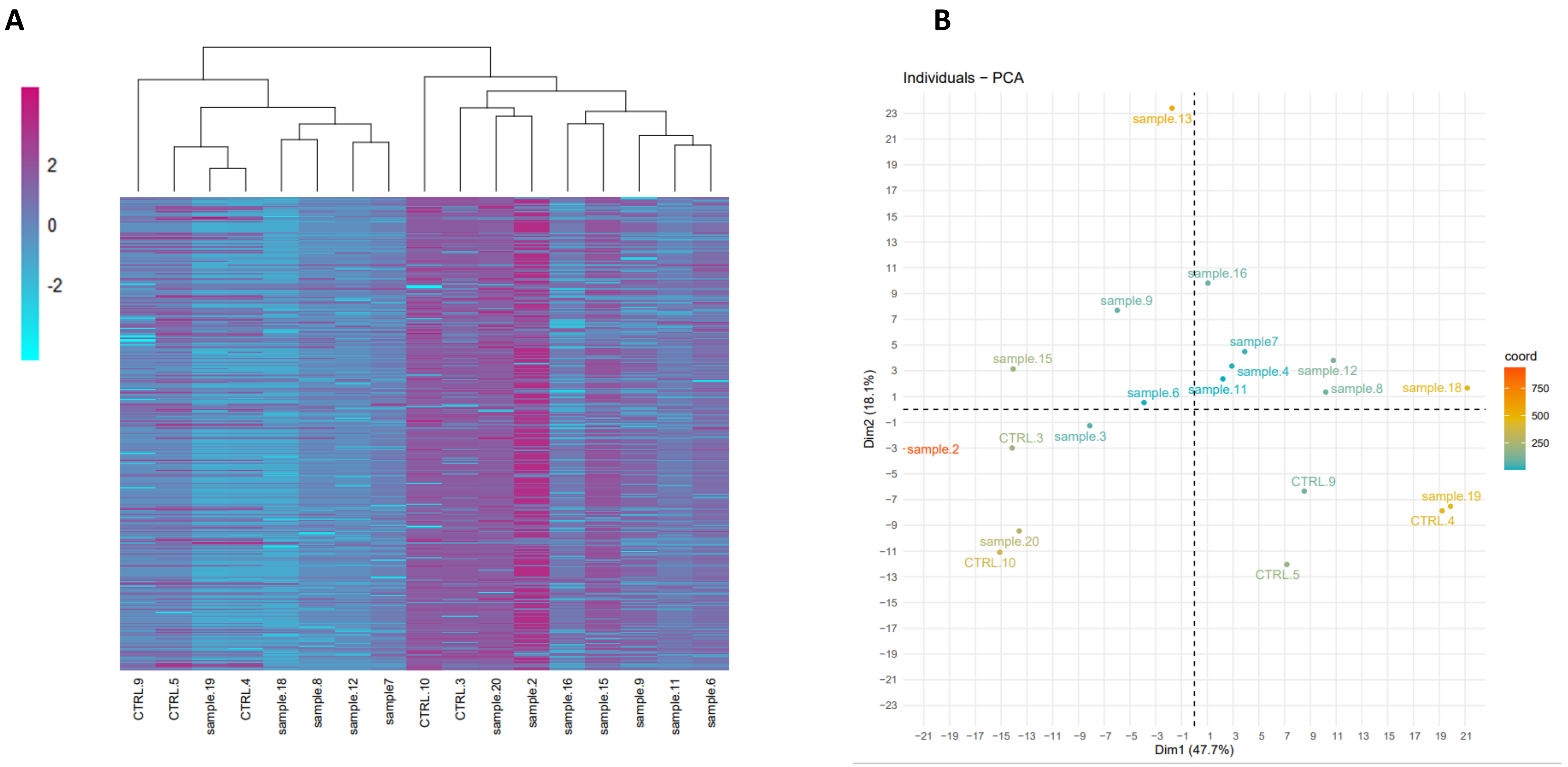

Supplementary Figure 2 A Heat map diagram shows cluster analysis of differentially expressed miRNAs . The colors represent the expression values of differentially expressed miRNAs. Sample names are listed in the horizontal axis. . **B** PCA Analysis of differentially expressed miRNAs in severe female AIS patients and female HC.
