# Supplementary material for: Investigating the Differential Circulating microRNA Expression in Adolescent Females with Severe Idiopathic Scoliosis: A Proof-of-Concept Observational Clinical Study
Source: Int J Mol Sci. 2024 Jan 1;25(1):570. doi: 10.3390/ijms25010570 (PMC10779108; doi:10.3390/ijms25010570)
Supplement: Supplementary file 1 [file ijms-25-00570-s001.zip › Supplementary Figure 3 REVISED.pdf]

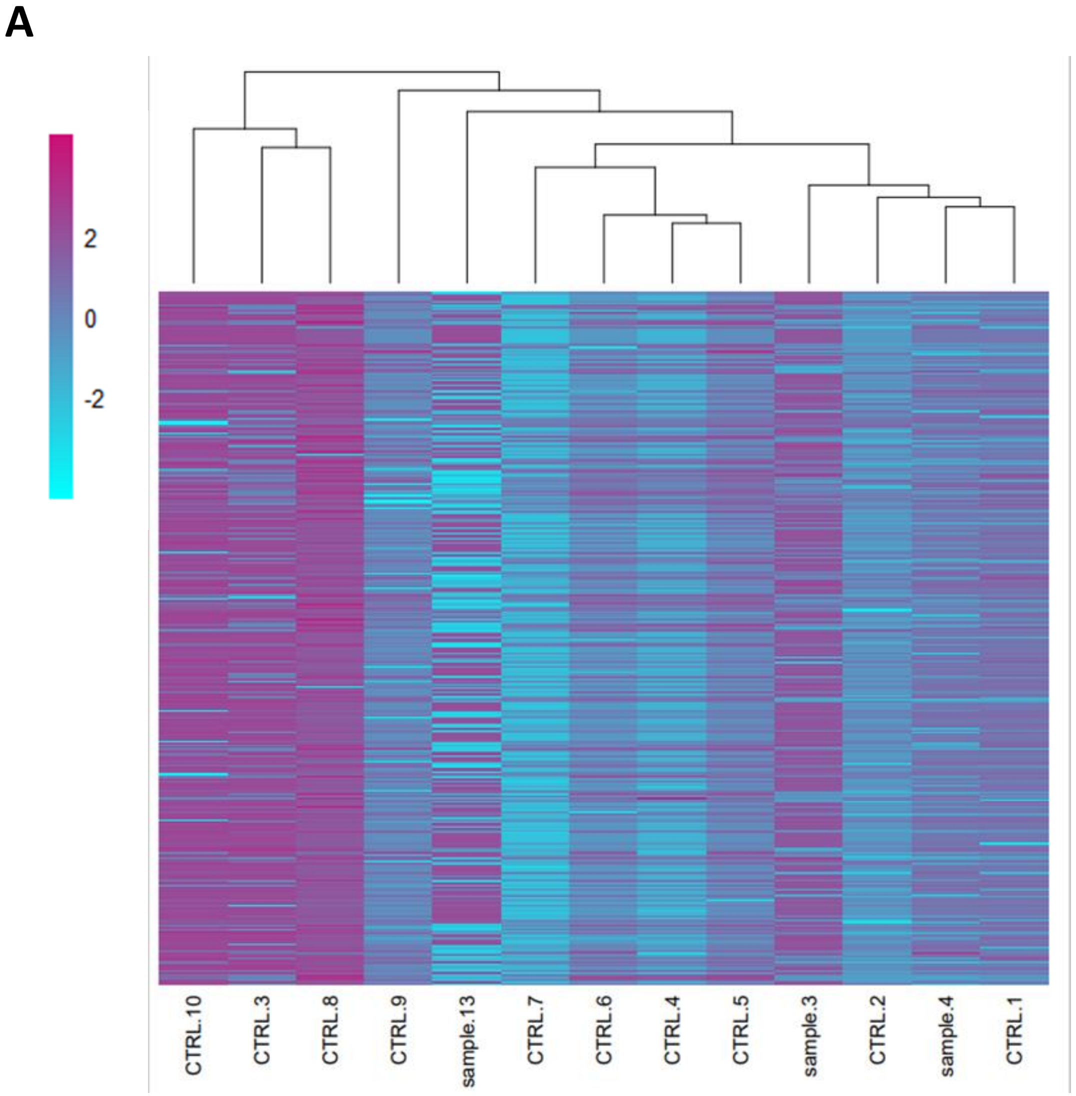

Heat map analysis of circulating miRNAs in moderate AIS vs HC

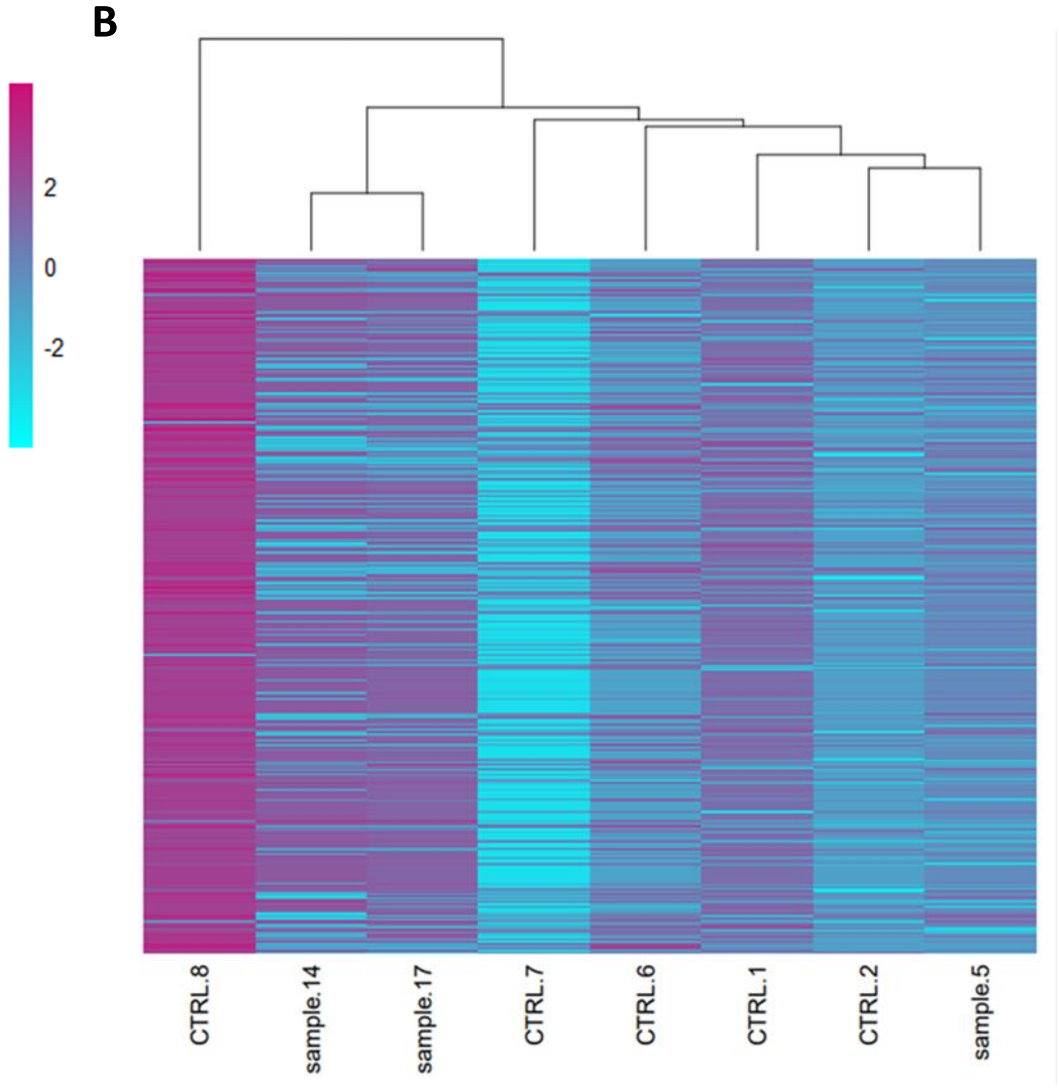

Heat map analysis of circulating miRNAs in severe male AIS vs males HC

Supplementary Figure 3 Heat map diagram shows cluster analysis of differentially expressed miRNAs . The colors represent the expression values of differentially expressed miRNAs. Sample names are listed in the horizontal axis. Axis represents the clustering of miRNAs.

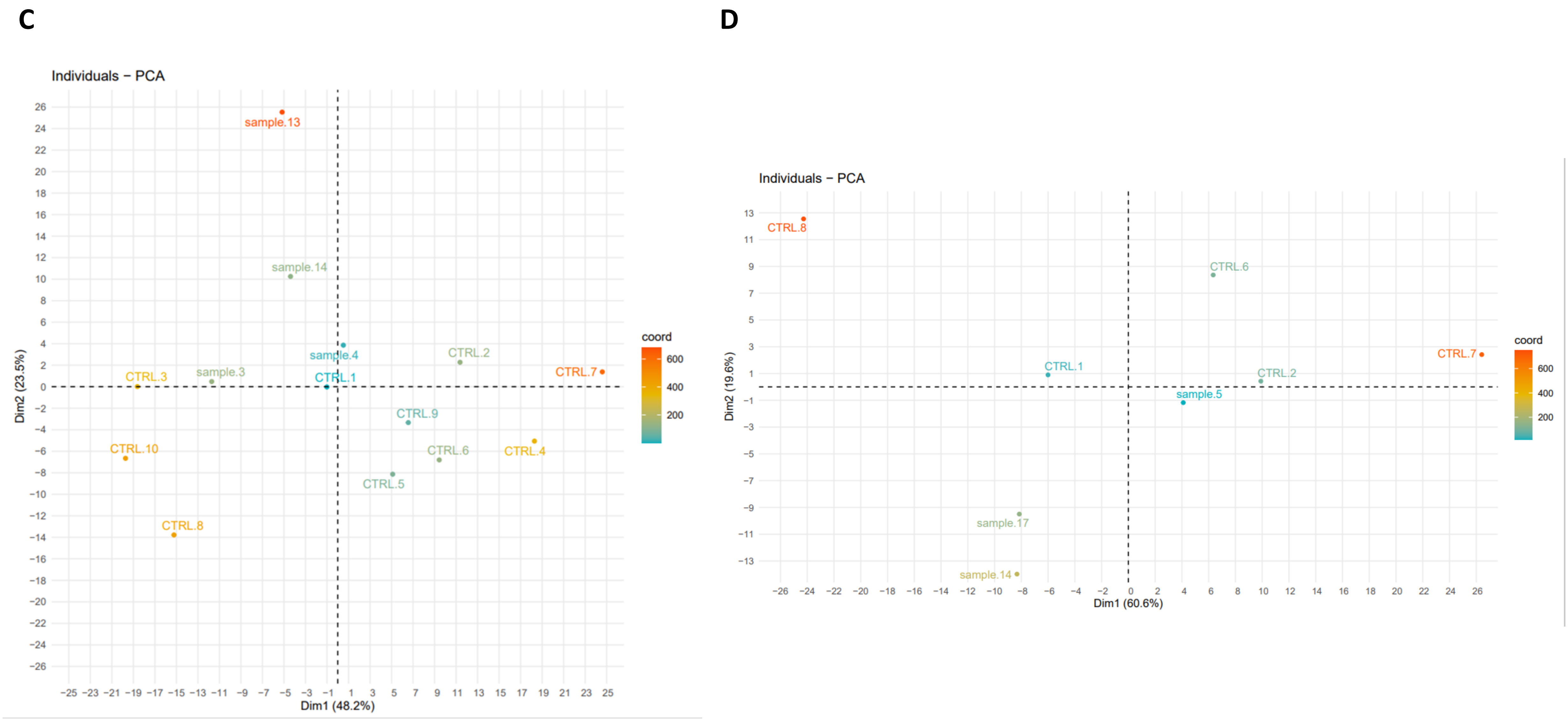

Supplementary Figure 2. PCA_plot analysis of differentially expressed miRNAs in moderate AIS vs HC (C) and in severe male AIS vs males HC (D).
