# Supplementary material for: Investigating the Differential Circulating microRNA Expression in Adolescent Females with Severe Idiopathic Scoliosis: A Proof-of-Concept Observational Clinical Study
Source: Int J Mol Sci. 2024 Jan 1;25(1):570. doi: 10.3390/ijms25010570 (PMC10779108; doi:10.3390/ijms25010570)
Supplement: Supplementary file 1 [file ijms-25-00570-s001.zip › Supplementary Figure 4.pdf]

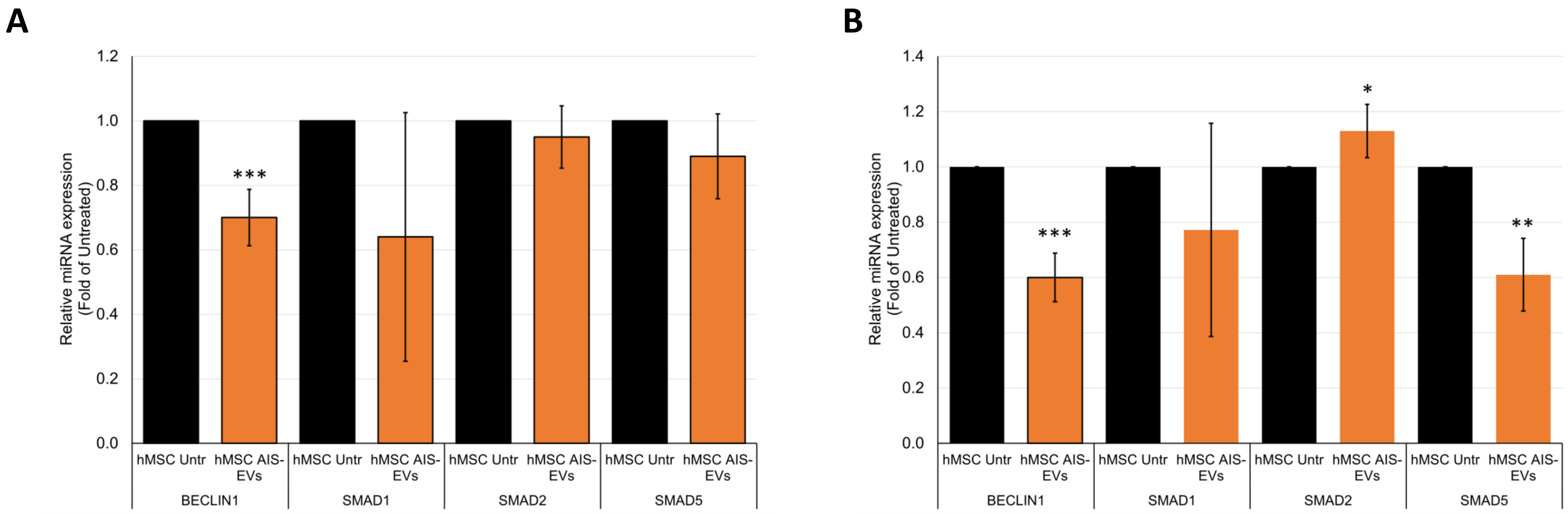

SF4. AIS-EVs affect expression of genes related to miR-30 family members. A qRT–PCR analysis of BECLIN1, SMAD1-2-5 in hMSCs untreated or treated with AIS-Evs in basal medium. **B** qRT–PCR analysis of BECLIN1, SMAD1-2-3 in hMSCs untreated or treated with AIS-Evs in osteogenic medium.
