# Supplementary material for: Investigating the Differential Circulating microRNA Expression in Adolescent Females with Severe Idiopathic Scoliosis: A Proof-of-Concept Observational Clinical Study
Source: Int J Mol Sci. 2024 Jan 1;25(1):570. doi: 10.3390/ijms25010570 (PMC10779108; doi:10.3390/ijms25010570)
Supplement: Supplementary file 1 [file ijms-25-00570-s001.zip › Supplementary Table 1.pdf]

Gender	Age	Lenke classification system	Location of Curve		Cobb		Risser	BMI
			Apex 1	Apex 2 (double scoliotic curve)	Cobb 1	Cobb 2 (double scoliotic curve)		
F	15	Lenke 1A	T10	/	60	/	2	19.05
F	17	Lenke 1C	T10	/	92	/	5	19.23
F	11	Lenke 1A	T9	/	62	/	3	17.12
F	13	Lenke 1C	T9	/	61	/	2	18.75
F	14	Lenke 6	T9	L2	71	80	5	24.14
F	15	Lenke 1A	T11	/	21	/	4	33.20
F	15	Lenke 1C	T7	/	63	/	4	24.17
F	15	Lenke 1A	T8	/	57	/	5	28.83
F	15	Lenke 1A	T10	/	45	/	5	18.61
F	14	Lenke 1C	T7	/	51	/	2	24.65
F	13	Lenke 1C	T9	/	81	/	3	18.80
F	16	Lenke 1A	T8	/	62	/	5	21.67
F	15	Lenke 1B	T8	/	50	/	4	26.64
F	15	Lenke 3	T12	T6	24	/	5	18.22
F	12	Lenke 2A	T5	T10	30	32	0	19.29
F	14	Lenke 5	L1/L2	/	65	/	0	20.80
F	16	Lenke 5	L2	/	32	/	0	16.40
M	16	Lenke 1C	T9	/	73	/	5	26.03
M	15	Lenke 1A	T6	/	45	/	4	19.35
M	17	Lenke 1A	T9	/	47	/	4	16.44

Supplementary Table 1. Demographic and clinical data of 20 AIS patients.
