# Supplementary material for: Investigating the Differential Circulating microRNA Expression in Adolescent Females with Severe Idiopathic Scoliosis: A Proof-of-Concept Observational Clinical Study
Source: Int J Mol Sci. 2024 Jan 1;25(1):570. doi: 10.3390/ijms25010570 (PMC10779108; doi:10.3390/ijms25010570)
Supplement: Supplementary file 1 [file ijms-25-00570-s001.zip › Supplementary Table 2.pdf]

miRNA	p_value	log2_fold_change	fold_change
**hsa-miR-425**	0.00001	5.95578	62.06791
**hsa-miR-30e-3p**	0.00019	5.70813	52.27787
**dme-miR-7**	0.00038	5.38914	41.90763
**hsa-miR-1303**	0.00044	5.01863	32.41581
**hsa-miR-126**	0.00051	6.28777	78.12830
**hsa-miR-30a-5p**	0.00063	6.81561	112.64240
**hsa-miR-26b-**	0.00068	4.58629	24.02214
**hsa-miR-30d**	0.00072	5.68944	51.60504
**hsa-miR-942**	0.00073	5.99987	63.99408
**hsa-miR-223**	0.00075	5.63184	49.58531
**hsa-miR-1249**	0.00138	4.53433	23.17227
**hsa-miR-30a-3p**	0.00164	5.01053	32.23438
**hsa-miR-18a**	0.00185	4.27564	19.36853
**hsa-miR-1180**	0.00208	4.79986	27.85498
**hsa-miR-766**	0.00217	6.01353	64.60284
**hsa-miR-625**	0.00309	4.98292	31.62343
**hsa-miR-320b**	0.00320	5.43490	43.25817
**hsa-miR-144**	0.00350	4.55020	23.42858
**hsa-miR-378**	0.00350	4.05381	16.60807
**hsa-miR-106b**	0.00395	5.30487	39.52971
**hsa-miR-22-**	0.00414	4.65593	25.21016
**hsa-miR-99b**	0.00434	3.70923	13.07943
**hsa-miR-151-3p**	0.00455	5.05364	33.21207
**hsa-miR-628-3p**	0.00475	4.08128	16.92727
**hsa-miR-1227**	0.00481	3.81425	14.06704
**hsa-miR-151-5p**	0.00599	3.78961	13.82885
**hsa-miR-663b**	0.00642	3.92686	15.20902
**hsa-miR-572**	0.00652	3.45350	10.95490
**hsa-miR-744**	0.00702	3.58582	12.00712
**hsa-miR-335**	0.00706	4.86174	29.07571
**hsa-miR-1274a**	0.00724	4.55393	23.48921
**rno-miR-7**	0.00732	4.73763	26.67889
**hsa-miR-409-3p**	0.00783	5.56364	47.29583
**hsa-miR-340**	0.00790	4.14238	17.65961
**hsa-miR-93**	0.00829	5.47608	44.51055
**hsa-miR-200a**	0.00879	-3.35707	0.08416
**hsa-miR-1260**	0.00925	5.37743	41.56883
**hsa-miR-1255b**	0.00967	3.35694	10.24568
**hsa-miR-144**	0.00972	3.58360	11.98869
**hsa-miR-629**	0.01005	3.92036	15.14068
**hsa-miR-25**	0.01018	3.70772	13.06578
**hsa-miR-661**	0.01052	5.09521	34.18301
**hsa-miR-183**	0.01107	3.57883	11.94908
**hsa-miR-1285**	0.01345	2.83266	7.12385
**hsa-miR-454**	0.01375	3.19269	9.14315
**hsa-miR-1225-3p**	0.01680	4.59359	24.14401

**hsa-miR-1276**	0.01724	2.99307	7.96168
**hsa-miR-1294**	0.02043	-7.75431	0.00536
**hsa-miR-432**	0.02275	4.28440	19.48648
**hsa-miR-1226**	0.02604	3.66240	12.66169
**hsa-miR-720**	0.02703	2.89337	7.43006
**hsa-miR-1247**	0.02788	2.94736	7.71338
**hsa-miR-378-1**	0.02825	3.46199	11.01952
**hsa-miR-638**	0.02888	3.36970	10.33667
**hsa-miR-191**	0.03388	3.02204	8.12317
**hsa-miR-664**	0.03642	3.34842	10.18531
**hsa-miR-505**	0.03716	2.49286	5.62894
**hsa-miR-1290**	0.03933	2.78569	6.89567
**hsa-miR-564**	0.04328	4.62348	24.64938
**hsa-miR-648**	0.04362	3.35770	10.25108
**hsa-miR-548m**	0.04628	-3.36354	0.08047
**hsa-miR-1270**	0.04688	2.71617	6.57128
**hsa-miR-136**	0.04921	2.85091	7.21453

Supplementary Table 2. Circulating miRNAs up- and down-regulated in plasma of AIS patients versus plasma of HC.
