# Supplementary material for: Investigating the Differential Circulating microRNA Expression in Adolescent Females with Severe Idiopathic Scoliosis: A Proof-of-Concept Observational Clinical Study
Source: Int J Mol Sci. 2024 Jan 1;25(1):570. doi: 10.3390/ijms25010570 (PMC10779108; doi:10.3390/ijms25010570)
Supplement: Supplementary file 1 [file ijms-25-00570-s001.zip › Supplementary Table 3.pdf]

miRNA	p_value	log2_fold_change	fold_change
**hsa-miR-425-**	0.00026	5.53327	46.31049
**hsa-miR-30e-3p**	0.00105	5.42657	43.00898
**hsa-miR-99b-**	0.00201	4.42454	21.47435
**hsa-miR-1276**	0.00218	4.77019	27.28785
**hsa-miR-30a-5p**	0.00242	7.02147	129.91941
**hsa-miR-320B**	0.00245	6.16202	71.60667
**hsa-miR-572**	0.00274	4.32011	19.97480
**dme-miR-7**	0.00327	5.42253	42.88879
**hsa-miR-30d**	0.00357	5.96757	62.57740
**hsa-miR-1274A**	0.00375	5.79629	55.57208
**hsa-miR-30a-3p**	0.00375	5.28395	38.96064
**hsa-miR-1180**	0.00406	5.49635	45.14056
**hsa-miR-26b-**	0.00413	4.19820	18.35626
**hsa-miR-1249**	0.00527	5.89403	59.46733
**hsa-miR-520D-3P**	0.00549	4.21890	18.62154
**hsa-miR-223--**	0.00630	5.33710	40.42275
**hsa-miR-571**	0.00632	7.61241	195.68718
**hsa-miR-126-**	0.00634	5.97114	62.73251
**hsa-miR-1183**	0.00694	7.18857	145.87338
**hsa-miR-663B**	0.00719	5.51693	45.78917
**hsa-miR-629**	0.00758	4.27026	19.29644
**hsa-miR-564**	0.00770	7.78190	220.08197
**hsa-miR-942**	0.00788	5.77996	54.94676
**hsa-miR-151-3p**	0.00798	5.33781	40.44279
**hsa-miR-638**	0.00884	4.86144	29.06954
**hsa-miR-144**	0.00962	5.59373	48.29266
**hsa-miR-648**	0.00984	4.16855	17.98290
**hsa-miR-144-**	0.01222	4.01809	16.20190
**hsa-miR-1294**	0.01387	-13.24190	0.00010
**hsa-miR-378**	0.01679	4.43655	21.65382
**hsa-miR-200a-**	0.02323	-4.38806	0.04776
**hsa-miR-765**	0.02341	-13.24643	0.00010
**hsa-miR-1298**	0.02372	9.09901	548.37083
**hsa-miR-1303**	0.02498	4.80421	27.93898
**hsa-miR-1260**	0.02500	6.65283	100.62397
**hsa-miR-661**	0.02853	6.23498	75.32105
**hsa-miR-378-1**	0.03078	3.88078	14.73098
**hsa-miR-573**	0.03530	6.01546	64.68945
**hsa-miR-639**	0.03664	7.20238	147.27582
**hsa-miR-624**	0.03855	4.69861	25.96708
**hsa-miR-93-**	0.03865	4.85814	29.00329
**hsa-miR-892b**	0.03952	4.43710	21.66202
**hsa-miR-1233**	0.04018	5.26918	38.56406
**hsa-miR-766**	0.04031	4.53372	23.16259
**hsa-miR-720**	0.04051	3.18187	9.07484
**hsa-miR-340-**	0.04859	3.70258	13.01934

**hsa-miR-744-**	0.04968	3.43667	10.82785

Supplementary Table 3. Circulating miRNAs up- and down-regulated in plasma of severe female AIS patients versus plasma of female HC.
