# Supplementary figures and images for: Investigating the Differential Circulating microRNA Expression in Adolescent Females with Severe Idiopathic Scoliosis: A Proof-of-Concept Observational Clinical Study
Source: Int J Mol Sci. 2024 Jan 1;25(1):570. doi: 10.3390/ijms25010570 (PMC10779108; doi:10.3390/ijms25010570)

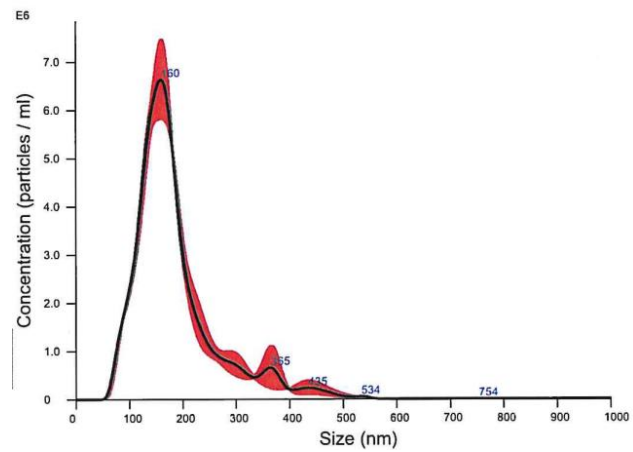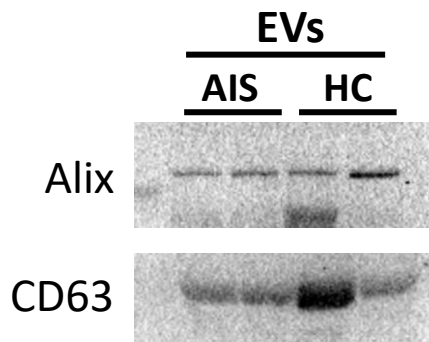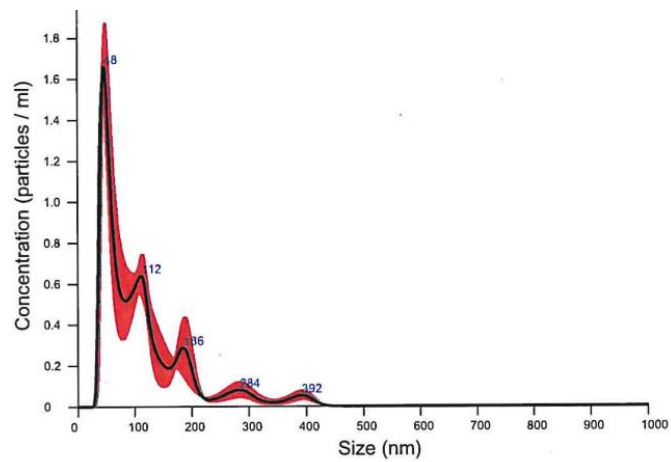

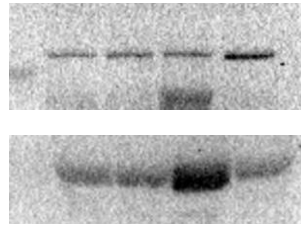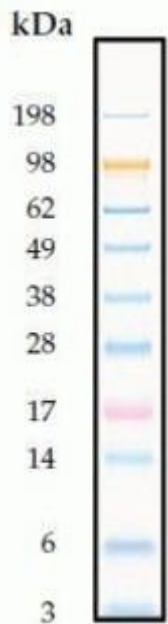

CD63 53 KDa

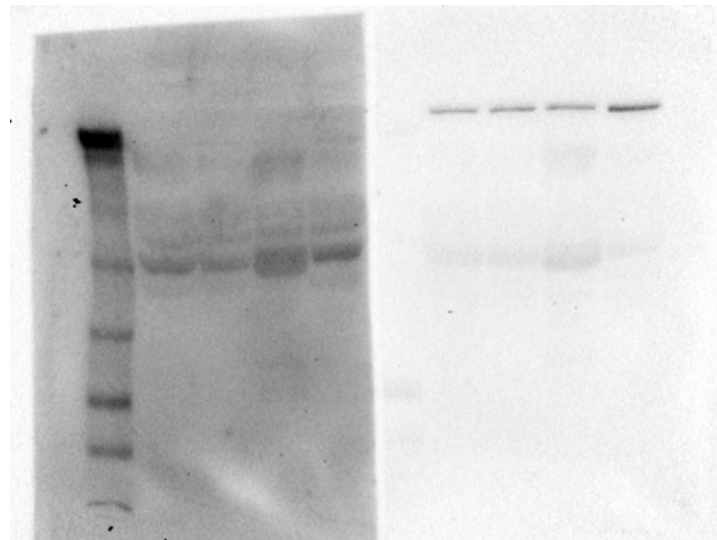

Alix 95kDa

Supplement: Supplementary file 1 [file ijms-25-00570-s001.zip › WB AIS EVs.pdf]
